# Expert opinion on NSCLC small specimen biomarker testing — Part 2: Analysis, reporting, and quality assessment

**DOI:** 10.1007/s00428-022-03344-1

**Published:** 2022-07-20

**Authors:** Frédérique Penault-Llorca, Keith M. Kerr, Pilar Garrido, Erik Thunnissen, Elisabeth Dequeker, Nicola Normanno, Simon J. Patton, Jenni Fairley, Joshua Kapp, Daniëlle de Ridder, Aleš Ryška, Holger Moch

**Affiliations:** 1 University Clermont Auvergne, INSERM U1240, Centre Jean Perrin, Clermont-Ferrand, France; 2 Department of Pathology, Aberdeen University Medical School and Aberdeen Royal Infirmary, Aberdeen, UK; 3 Medical Oncology Department, Hospital Universitario Ramón Y Cajal, University of Alcalá, Madrid, Spain; 4 Amsterdam University Medical Center, VU Medical Center, Amsterdam, the Netherlands; 5 Department of Public Health, Biomedical Quality Assurance Research Unit, Campus Gasthuisberg, University Leuven, Leuven, Belgium; 6 Cell Biology and Biotherapy Unit, Istituto Nazionale Tumori “Fondazione Giovanni Pascale” IRCCS, Naples, Italy; 7 EMQN CIC, Manchester, UK; 8 GenQA, Edinburgh, UK; 9 Amgen (Europe) GmbH, Rotkreuz, Switzerland; 10 Amgen BV, Breda, the Netherlands; 11 Department of Pathology, Charles University Medical Faculty Hospital, Hradec Králové, Czech Republic; 12 Department of Pathology and Molecular Pathology, University Hospital Zurich and University of Zurich, Zurich, Switzerland

**Keywords:** Best practice, External quality assessment, Liquid biopsy, Molecular diagnostics, Next-generation sequencing, Non-small cell lung carcinoma

## Abstract

The diagnostic work-up for non-small cell lung cancer (NSCLC) requires biomarker testing to guide therapy choices. This article is the second of a two-part series. In Part 1, we summarised evidence-based recommendations for obtaining and processing small specimen samples (i.e. pre-analytical steps) from patients with advanced NSCLC. Here, in Part 2, we summarise evidence-based recommendations relating to analytical steps of biomarker testing (and associated reporting and quality assessment) of small specimen samples in NSCLC. As the number of biomarkers for actionable (genetic) targets and approved targeted therapies continues to increase, simultaneous testing of multiple actionable oncogenic drivers using next-generation sequencing (NGS) becomes imperative, as set forth in European Society for Medical Oncology guidelines. This is particularly relevant in advanced NSCLC, where tissue specimens are typically limited and NGS may help avoid tissue exhaustion compared with sequential biomarker testing. Despite guideline recommendations, significant discrepancies in access to NGS persist across Europe, primarily due to reimbursement constraints. The use of increasingly complex testing methods also has implications for the reporting of results. Molecular testing reports should include clinical interpretation with additional commentary on sample adequacy as appropriate. Molecular tumour boards are recommended to facilitate the interpretation of complex genetic information arising from NGS, and to collaboratively determine the optimal treatment for patients with NSCLC. Finally, whichever testing modality is employed, it is essential that adequate internal and external validation and quality control measures are implemented.

## Introduction

### Who should we test?

Biomarker testing is now essential for guiding treatment decisions in advanced non-small cell lung cancer (NSCLC), with European Society for Medical Oncology (ESMO) guidelines suggesting that “all patients with advanced, possible, probable or definite, adenocarcinoma should be tested for oncogenic drivers” [1]. Additionally, molecular testing is recommended in cohorts of patients with non-adenocarcinoma histology (e.g. squamous cell carcinoma) who are < 50 years of age [2] and those who are never-smokers, long-time ex-smokers, or light-smokers (< 15 pack-years) [1]. This strategy is driven by the relative probability of finding an actionable alteration. As noted in Part 1 [3], there is a growing body of evidence indicating that patients with actionable oncogenic driver mutations who receive targeted therapy have improved clinical outcomes versus those without actionable driver mutations who receive chemotherapy [4–6].

### Which biomarkers should we test?

The clinical armamentarium for advanced NSCLC currently comprises seven European Medicines Agency (EMA)–approved targeted agents with associated biomarkers (excluding programmed death ligand 1 [PD-L1]; see Table 1) [7, 8]. These biomarkers for actionable genetic targets now include sensitising mutations in exons 18, 19, and 21 of the epidermal growth factor receptor (*EGFR*), Kirsten rat sarcoma viral oncogene homolog (*KRAS*) *p.G12C* point mutation, B-Raf proto-oncogene V600E point mutation (*BRAF p.V600E*), and rearrangements involving anaplastic lymphoma kinase (*ALK*), ROS proto-oncogene 1 (*ROS1*), neurotrophic tyrosine receptor kinase (*NTRK1*, *2*, and *3*), and rearranged during transfection (*RET*) [7, 8]. As noted in ESMO guidelines, testing for *EGFR* mutations and rearrangements involving *ALK* and *ROS1* is now considered mandatory in most European countries [1]. As first-line B-Raf/mitogen-activated protein kinase (MEK) inhibitors become more widely approved, *BRAF p.V600E* mutation testing is also mandated in many oncology services [1]. *KRAS p.G12C* is now an actionable genetic target in Europe following approval of sotorasib by the European Commission in January 2022 [9]. *NTRK* is a target with approved treatments in many European countries, while the Erb-B2 receptor tyrosine kinase 2/human epidermal growth factor receptor 2 (*ERBB2/HER2*) and hepatocyte growth factor receptor (*MET*) exon 14 skipping mutations are evolving targets/biomarkers [1]. An ESMO Precision Medicine Working Group developed the ESMO Scale for Clinical Actionability of molecular Targets (ESCAT) to help clinicians prioritise actionability of the various genetic targets [10]. An ESCAT level I alteration means that a drug has been validated in clinical trials and, therefore, the alteration should drive treatment decisions in daily clinical practice. ESMO recommends that all level I alterations are profiled in patients with lung adenocarcinoma using next-generation sequencing (NGS).

**Table 1:** Established and emerging biomarkers for NSCLC in Europe [7, 8]

Predictive biomarkers	Estimated frequency in NSCLC adenocarcinoma^e^	Guideline-recommended testing technologies	EMA-approved targeted therapy^h^
*EGFR* mutations^a^	15%^f^	Any appropriate, validated technology, subject to external quality assessment	Afatinib, dacomitinib, erlotinib, gefitinib, osimertinib
*KRAS p.G12C* mutations	13% 25–33% (all *KRAS* mutations)	PCR; pyrosequencing; NGS	Sotorasib^i^
*ALK* rearrangements^a^	5%	FISH (historical standard); IHC (validated against FISH); NGS^g^	Alectinib, brigatinib, ceritinib, crizotinib, lorlatinib
*ROS1* rearrangements^a^	2%	FISH (trial-validated standard); IHC to select for confirmatory FISH; NGS^g^	Crizotinib, entrectinib
*NTRK* rearrangements^a^	< 1%	IHC; FISH; PCR; NGS	Entrectinib, Iarotrectinib
*BRAF* mutations^b^	2%	Any appropriate, validated technology, subject to external quality assessment	Dabrafenib, trametinib
*RET* rearrangements	2%	Any validated test (e.g. FISH; PCR; NGS)	Selpercatinib
PD-L1 expression levels^c^	≥ 50% TPS: 33% 1–49% TPS: 30% < 1% TPS: 37%	IHC	Immune checkpoint inhibitors (pembrolizumab, nivolumab, atezolizumab, cemiplimab) alone or with chemotherapy
Emerging biomarkers^d^	Estimated frequency in NSCLC adenocarcinoma	Potential testing technology	Targeted therapies under investigation
*MET* exon skipping mutations	3%	IHC; FISH; NGS	Cabozantinib, capmatinib^j,k^, crizotinib, MGCD265, tepotinib^j,l,m^
*ERBB2/HER2* mutations and amplifications	2%	NGS	Ado-trastuzumab emtansine, afatinib, dacomitinib, fam-trastuzumab deruxtecan-nxki^k,j^, trastuzumab, mobocertinib
*NRG1* rearrangements	< 1%	NGS^g^	Afatinib, GSK2849330, AMG 888, seribantumab, zenocutuzumab
FGFR1	Data not available	NGS^g^	BGJ398, rogaratinib

Table adapted from Kerr et al. [8]. Copyright © 2021 The Authors. Published by Elsevier B.V. All rights reserved. Reproduced under the terms of Creative Commons Attribution 4.0 International (CC BY 4.0) license

*ALK* anaplastic lymphoma kinase, *BRAF* B-Raf proto-oncogene, *EGFR* epidermal growth factor receptor, *EMA* European Medicines Agency, *ERBB2* Erb-B2 receptor tyrosine kinase 2, *FDA* Food and Drug Administration, *FGFR1* fibroblast growth factor receptor-1, *FISH* fluorescence in situ hybridisation, *HER2* human epidermal growth factor receptor 2, *IHC* immunohistochemistry, *KRAS* Kirsten rat sarcoma viral oncogene homolog, *MEK* mitogen-activated protein kinase, *MET* hepatocyte growth factor receptor, *NGS* next-generation sequencing, *NRG1* neuregulin-1, *NSCLC* non-small cell lung cancer, *NTRK* neurotrophic tyrosine receptor kinase, *PD-L1* programmed cell death ligand 1, *RET* rearranged during transfection, *ROS1* ROS proto-oncogene 1, *PCR* polymerase chain reaction, *TPS* tumour proportion score

^a^Predicts response to targeted therapy with tyrosine kinase inhibitors

^b^Predicts response to BRAF with/without MEK inhibitors

^c^Predicts response to immunotherapy

^d^Under investigation as predictive biomarkers with the goal of identifying appropriate therapies for patients

^e^No specific driver known in over one-third of cases

^f^Exon 19 deletions, exon 21 *p.L858R* mutations, and exon 20 insertions comprise approximately 10%, 6%, and 2.5% of all mutations, respectively

^g^Emerging technology

^h^As of January 2022

^i^Other direct KRAS.^G12C^ inhibitors are in the pipeline, including adagrasib (MRTX849; FDA Breakthrough Therapy designation), GDC-6036, JNJ-74699157, JDQ443, LY3537982, D-1553

^j^FDA approval

^k^Approved in Japan

^l^Under review by EMA

^m^Approved in the UK under the Early Access to Medicine Scheme

In addition to the biomarkers for actionable genetic targets, PD-L1 expression by immunohistochemistry (IHC) is mandatory to inform treatment selection with immune checkpoint inhibitors (Table 1) [1]. ESMO guidelines specify a mandatory threshold of a tumour proportion score of ≥ 50% in first-line treatment [1]; the tumour proportion score is defined as the number of PD-L1 + tumour cells divided by the total number of viable tumour cells, multiplied by 100% [11]. However, definitions and thresholds for biomarker analyses are not standardised; in the case of PD-L1, at least five assays are available that have specific scoring systems and tumour site indications [12]. Studies have indicated a high level of concordance in the results of some of these assays for NSCLC [13]. Nevertheless, assay standardisation for emerging biomarkers is a challenge that will require the coordinated efforts of all stakeholders to ensure the future success of biomarker-guided targeted therapy [14].

The number of EMA-approved biomarkers for actionable targets is set to increase over coming years, owing to a rich pipeline of targeted therapeutic agents. These include *MET* exon 14 skipping mutations and gene amplifications, *ERBB2/HER2* mutations and amplifications, neuregulin-1 (*NRG1*) rearrangements, fibroblast growth factor receptor 1 (*FGFR1*), and *EGFR* exon 20 insertions [8]. Agents targeting these genes are under investigation and some of these have already received approval in certain countries (see Table 1).

**Fig. 1:**
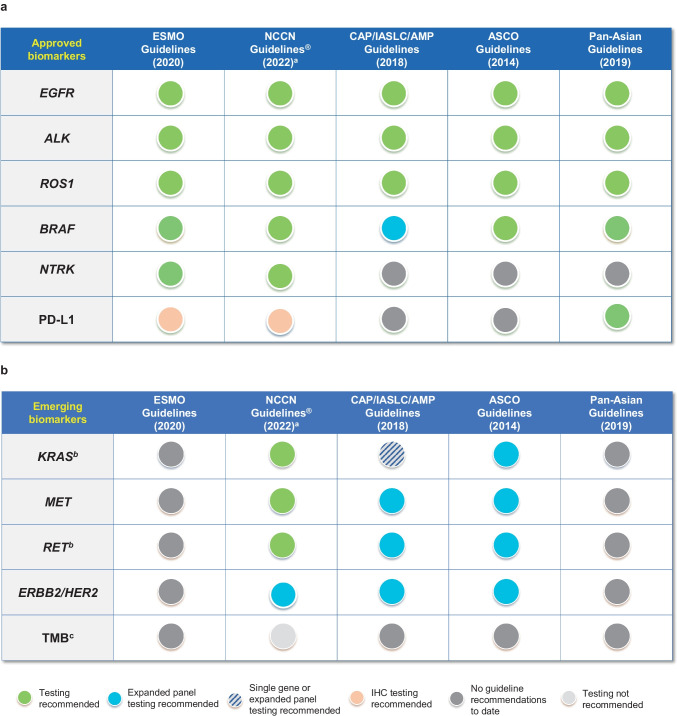
Summary of recommendations from international guidelines for **a** approved and **b** emerging biomarkers [8, 57]. Figure adapted from Kerr et al. [8]. Copyright © 2021 The Authors. Published by Elsevier B.V. All rights reserved. Reproduced under the terms of Creative Commons Attribution 4.0 International (CC BY 4.0) license. ^a^NCCN Clinical Practice Guidelines In Oncology (NCCN Guidelines^®^) for NSCLC provide recommendations for certain individual biomarkers that should be tested and recommend testing techniques but do not endorse any specific commercially available biomarker assays or commercial laboratories, ^b^biomarker testing for *KRAS* and *RET* is recommended in the NCCN Guidelines^®^, ^c^the NCCN Guidelines^®^ do not recommend TMB testing. *ALK* anaplastic lymphoma kinase, *AMP* Association for Molecular Pathology, *ASCO* American Society of Clinical Oncology, *BRAF* B-Raf proto-oncogene, *CAP* College of American Pathologists, *EGFR* epidermal growth factor receptor, *ERBB2* Erb-B2 receptor tyrosine kinase 2, *ESMO* European Society for Medical Oncology, *HER2* human epidermal growth factor receptor 2, *IASLC* International Association for the Study of Lung Cancer, *IHC* immunohistochemistry, *KRAS* Kirsten rat sarcoma viral oncogene homolog, *MET* hepatocyte growth factor receptor, *NCCN* National Comprehensive Cancer Network, *NTRK* neurotrophic tyrosine receptor kinase, *PD-L1* programmed cell death ligand 1, *RET* rearranged during transfection, *ROS1* ROS proto-oncogene 1, *TMB* tumour mutational burden

The rapid pace of innovation in targeted drug development, which is epitomised by the current and future state of precision oncology in NSCLC, makes it challenging for clinical guidelines and associated practice to keep pace. Figure 1 demonstrates the increasing gap between current recommendations and approved therapies across the main international guidelines for biomarker testing.

**Fig. 2:**
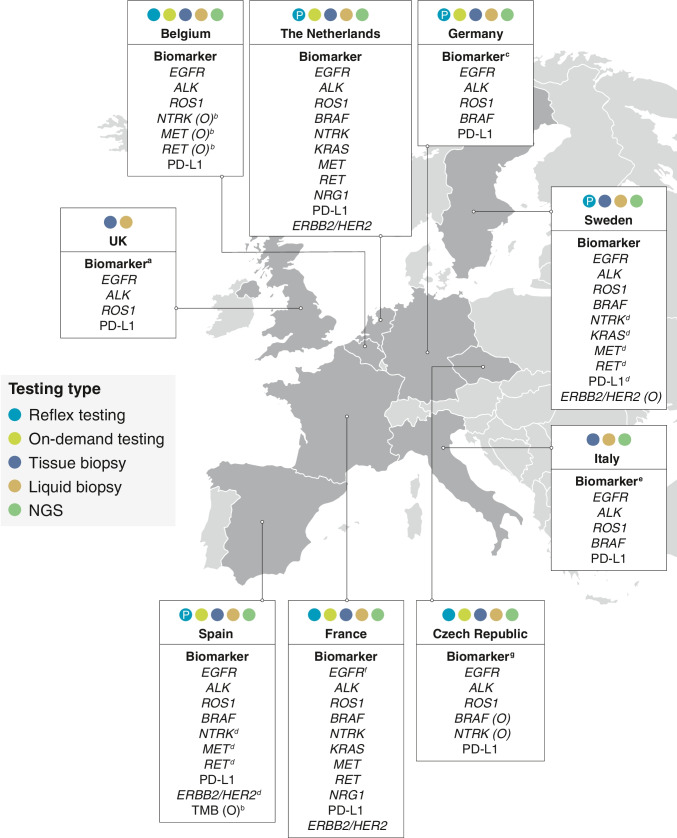
Summary of country-specific guidelines for biomarker testing of advanced or recurrent NSCLC [8]. Figure adapted from Kerr et al. [8]. Copyright © 2021 The Authors. Published by Elsevier B.V. All rights reserved. Reproduced under the terms of Creative Commons Attribution 4.0 International (CC BY 4.0) license. *ALK* anaplastic lymphoma kinase, *BRAF* B-Raf proto-oncogene, *EGFR* epidermal growth factor receptor, *ERBB2* Erb-B2 receptor tyrosine kinase 2, *HER2* human epidermal growth factor receptor 2, *KRAS* Kirsten rat sarcoma viral oncogene homolog, *MET* hepatocyte growth factor receptor, *NGS* next-generation sequencing, *NRG1* neuregulin-1, *NSCLC* non-small cell lung cancer, *NTRK* neurotrophic tyrosine receptor kinase, *O* optional, *P* preferred, *PD-L1* programmed cell death ligand 1, *RET* rearranged during transfection, *ROS1* ROS proto-oncogene 1, *TMB* tumour mutational burden. ^a^*NTRK* is also test-approved in limited circumstances; in England, some targeted therapies for other biomarkers may be available through the Cancer Drugs Fund. ^b^Consider other molecular tests, depending on clinic or drug availability. ^c^*NTRK*, *KRAS*, *MET*, *RET*, and *ERBB2*/*HER2* will be included in the current revision. ^d^The use of these biomarkers as individual tests is currently not indicated; instead, it is advised to include them in extended panels performed either initially in all advanced NSCLCs or when previous *EGFR*/*ALK*/*ROS1*/*BRAF* testing is negative. ^e^Liquid biopsy testing is recommended if the patient cannot undergo biopsy or if tissue molecular analysis results are uninformative. ^f^Liquid biopsy for *EGFR* assessment only when tissue biopsy is not available. ^g^On-demand testing for cases not fulfilling the reflex criteria (e.g. for squamous carcinomas with some suggestive clinical features [young age, non-smoker, etc.])

The gap between real-world practice and technical innovation is further increased by variation in national guidelines and reimbursement decisions, which often differ considerably from the initial EMA approvals in terms of timelines and outcomes. The significant variability between European countries in terms of real-world biomarker testing practice was illustrated in a recent review by Kerr and colleagues [8] (Fig. 2), highlighting that implementation of biomarker testing for patients with NSCLC continues to be suboptimal across certain regions and countries.

### How should we assess biomarkers in NSCLC?

The availability of seven EMA-approved, biomarker-directed NSCLC therapies (excluding checkpoint inhibitors) and emerging targeted therapies suggests an imperative for multiplexed, massively paralleled sequencing technology (i.e. NGS) over multiple single-gene tests as the standard of care for patients with advanced NSCLC. NGS enables simultaneous testing of multiple oncogenic drivers [1, 2, 10] and provides a method to cope with increasing numbers of actionable targets, and limited volumes of available tissue (as discussed in Part 1). NGS may allow the analysis of clinically relevant co-mutations such as serine/threonine kinase 11 (*STK11*), kelch-like ECH associated protein 1 (*KEAP1*) and tumour protein p53 (*TP53*), and DNA damage response pathway alterations involving breast cancer type 1/2 (*BRCA1/2*)*.* Other emerging predictors of neoantigen burden and immunotherapy response, such as tumour mutational burden (TMB), comprehensive genomic profiling (CGP), and DNA methylation, may also be analysed by NGS. As the number of evaluable biomarkers continues to increase, running multiple standalone assays in parallel or sequentially becomes increasingly inefficient in terms of time and cost, eventually tipping the balance in favour of NGS. Accordingly, recent ESMO guidelines state that the use of NGS for molecular testing is preferable for certain tumour types (e.g. level I alterations in lung adenocarcinoma, ovarian cancer, prostate cancer, and cholangiocarcinoma) [10], and NGS is rapidly being adopted as the standard approach to identify lung adenocarcinomas with oncogenic targets [1]. However, despite current guideline recommendations, there remain significant discrepancies in access to/use of NGS across Europe [15], where reimbursement constraints are a key limitation for adoption of best practice in biomarker testing.

In the previous article in this series, we explored the challenges and evidence-based recommendations related to obtaining sufficient quality tissue to undergo biomarker testing. In this review (Part 2), we summarise evidence-based recommendations relating to the analysis, reporting, and quality assessment of biomarker testing in small specimens from patients with advanced NSCLC. Where no guidelines or literature explicitly describe best practice, we report our recommendations for best practice according to the experience of the author group.

## Biomarker testing methodologies

### Single-gene or multiplex approaches?

Biomarker testing methodologies fall into two categories: single-gene or multiplex assays (i.e. NGS or multiplex polymerase chain reaction [PCR]) of DNA and/or RNA [1, 2]. Single-gene testing approaches include DNA sequencing by real-time quantitative PCR (qPCR), pyrosequencing or sanger sequencing, RNA sequencing by reverse transcriptase (RT)-PCR, detection of cell protein expression by IHC, and detection of gene fusions/amplifications by (fluorescence) in situ hybridisation ([F]ISH). The appropriate diagnostic modality depends on the molecular target of interest, as illustrated in Table 2. To cover testing of all the biomarkers in Table 2, broad-panel NGS sequencing is more cost-effective than multiple standalone biomarker tests using combinations of IHC, FISH, and PCR (acknowledging that IHC is currently the only reliable method for PD-L1 assessment and is the method of first choice for *ALK*, with equivocal results confirmed by FISH [1, 2]). In agreement with this expectation, studies have shown that NGS is more cost-effective than single-gene testing when multiple targets need to be tested [16–18], and increasing use of NGS versus single-gene testing correlates with an increase in life-years gained for patients with advanced NSCLC [18]. Overall, NGS represents an efficient alternative to single-gene testing [19].

**Table 2:** Recommended analytical methodology for current and emerging predictive biomarkers for NSCLC

Biomarker	Type	Analytical techniques
*EGFR* ex 18, 19, 21	Mutation	DNA-SEQ (PCR/NGS)
*KRAS p.G12C*	Mutation	DNA-SEQ (PCR/NGS)
*ALK*	Fusion	IHC & FISH, DNA-SEQ, RNA-SEQ (PCR/NGS)
*MET* exon 14 skipping	Mutation/rearrangement	DNA-SEQ (PCR/NGS)/RNA-SEQ/FISH
*EGFR* ex 20	Mutation	DNA-SEQ (PCR/NGS)
*BRAF p.V600E*	Mutation	DNA-SEQ (PCR/NGS)
*ERBB2/HER2*	Mutation	DNA-SEQ (PCR/NGS)
*RET*	Fusion	FISH, DNA-SEQ, RNA-SEQ (PCR/NGS)
*ROS1*	Fusion	IHC & FISH, DNA-SEQ, RNA-SEQ (PCR/NGS)
*NRG1*	Fusion	FISH, DNA-SEQ, RNA-SEQ (PCR/NGS)
*NTRK1, 2, 3*	Fusion	IHC & FISH, DNA- SEQ, RNA-SEQ (PCR/NGS)
PD-L1	Expression	IHC

*ALK* anaplastic lymphoma kinase, *BRAF* B-Raf proto-oncogene, *EGFR* epidermal growth factor receptor, *ERBB2* Erb-B2 receptor tyrosine kinase 2, *FISH* fluorescent in situ hybridisation, *HER2* human epidermal growth factor receptor 2, *IHC* immunohistochemistry, *KRAS* Kirsten rat sarcoma viral oncogene homolog, *MET* hepatocyte growth factor receptor, *NGS* next-generation sequencing, *NRG1* neuregulin-1, *NSCLC* non-small cell lung cancer, *NTRK* neurotrophic tyrosine receptor kinase, *PCR* polymerase chain reaction, *PD-L1* programmed cell death ligand 1, *RET* rearranged during transfection, *ROS1* ROS proto-oncogene 1, *SEQ* sequencing

### DNA or RNA?

While current DNA-based NGS can, theoretically, be used to detect sensitising mutations (point mutations, deletions, and insertions), copy number variations, and structural rearrangements (gene fusions), reliance on DNA-based fusion detection carries a risk of false negatives related to missing relevant fusions when large intronic regions stand between the fusion partners [20, 21]. The sensitivity of DNA-based NGS assays may be limited by the size of the intronic regions for genes such as *NTRK*, as the breakpoints usually occur within large intronic regions [20]. However, differences exist between the various systems used for library preparation in terms of the false negative error rate. In contrast to DNA, RNA sequencing is not affected by intronic regions that are spliced out during transcription. Therefore, the authors recommend using RNA-based NGS in parallel to DNA-based NGS to help improve sensitivity for the detection of gene fusions. The choice of the technology is also important, as hybrid capture assay and anchored multiplex technology allow broader fusion analysis but require a larger amount of material than amplicon-based methods [22]. Furthermore, RNA-based NGS allows identification of gene transcripts, permitting conclusions regarding in-frame gene fusions that are fully functioning, as well as the identification of gene fusion partners. In terms of the potential impact on available tissue, one-step co-extraction of RNA and DNA and simultaneous NGS of both DNA and RNA can help reduce tissue consumption [23, 24].

### Is there a role for IHC in the detection of gene fusions?

It should be acknowledged that, particularly in the context of fusion gene testing, IHC may be complementary to, and/or an alternative to, sequencing or FISH testing; however, in the authors’ experience, the expense and tissue consumption of these approaches should also be considered. Sometimes, elevations in protein levels are observed in tumour cells when driven by an oncogenic fusion gene. Detection of gene-product overexpression by IHC is a useful screening tool for assessing *ALK*, *ROS1*, and *NTRK* fusions in NSCLC. This approach is recommended in ESMO guidelines [1], and the US Food and Drug Administration has approved the Roche VENTANA ALK (D5F3) CDx IHC assay as a primary therapy-determining test for ALK kinase inhibitors [25]. For *ROS1* and *NTRK* IHC + cases, confirmation by another molecular method (e.g. FISH, qPCR, NGS) is mandatory [1]. For *RET* fusions, IHC is not recommended as a screening tool, as false positive and negative cases have been reported [26]. Taken together, combined DNA/RNA NGS, using appropriately validated assays and processed by suitably qualified operators, is a reliable and efficient approach for comprehensive detection of all approved and emerging biomarkers in advanced NSCLC (excluding PD-L1 detection by IHC). There may be other roles for IHC in the context of fusion gene testing. IHC may be possible in samples with few tumour cells or with high non-neoplastic cell contamination and where NGS fails or is not feasible. Strong IHC staining may be directly clinically actionable (e.g. for ALK fusions) or strongly indicative of the presence of a fusion gene (e.g. ROS1 and NTRK), in the appropriate histological context. There is also evidence that presence of the protein (positive IHC) may be indicative of greater probability of clinical response to therapy [27, 28], suggesting that IHC may be complementary to molecular methods for fusion gene identification/detection.

### Tissue or liquid biopsy?

Sequencing of plasma-circulating cell-free DNA (cfDNA) via liquid biopsy is a complementary approach to tissue-based biomarker testing, particularly when tissue samples are insufficient or unsuitable/inadequate for biomarker testing, or if re-biopsy cannot be performed safely [29]. In the authors’ experience, cfDNA sequencing analysis can be conducted using as little as 6 mL of peripheral whole blood stored at room temperature in ethylenediaminetetraacetic acid (EDTA) tubes. Ideally, blood collected in EDTA tubes requires centrifugation within 3 h (to reduce degradation of cfDNA and the risk of a false negative result), yielding 3 mL of plasma, which subsequently undergoes cfDNA extraction using commercially available kits. A variety of sequencing methods may then be applied to the extracted DNA including qPCR, droplet digital PCR, and NGS [30]. Analytical techniques must be highly sensitive to detect tumour-specific cfDNA, which represents only a small fraction of total circulating cfDNA.

While plasma is most commonly used for liquid biopsy, all biological fluids can potentially represent a source of tumour DNA for testing; however, limited data exist on the use of these alternative sources in the genomic characterisation of NSCLC for guiding therapy. Nevertheless, evidence suggests that cerebrospinal fluid testing may be more sensitive than that of plasma for detection of genomic alterations in patients with NSCLC and leptomeningeal metastases [31, 32]. It has also been suggested that the combination of plasma and urine testing can increase the sensitivity of *EGFR* mutation testing in NSCLC [33].

**Fig. 3:**
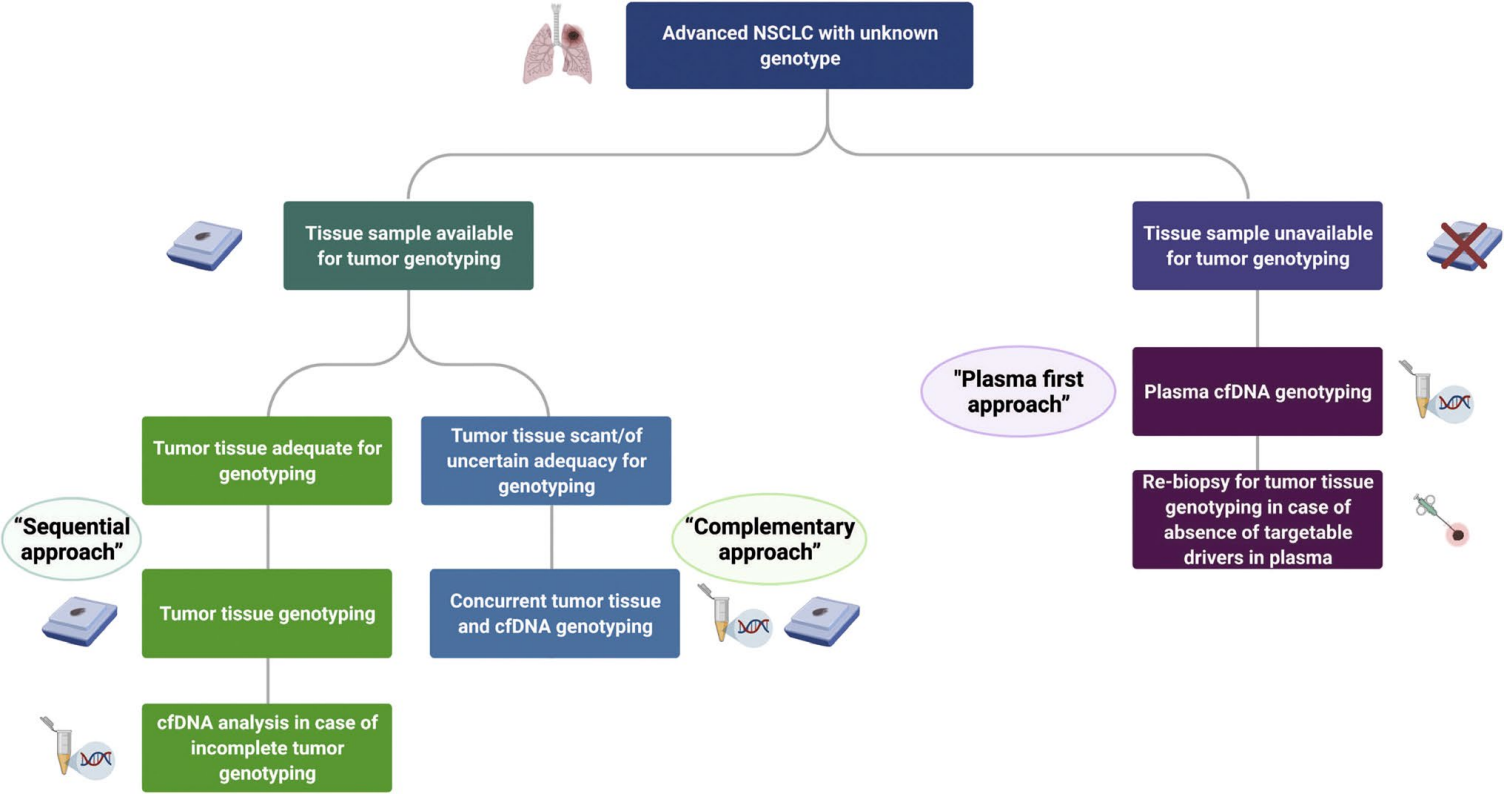
Diagnostic algorithm for liquid biopsy use in advanced/metastatic NSCLC (updated IASLC consensus statement) [34]. Figure reproduced from [34], J Thorac Oncol, Vol. 16, Rolfo C, et al., Liquid Biopsy for Advanced NSCLC: A Consensus Statement From the International Association for the Study of Lung Cancer, pages 1647–1622. Copyright (2021), with permission from J Thorac Oncol. Published by Elsevier Ltd. All rights reserved. Sequential approach: tissue followed by cfDNA complementary approach, concurrent tissue and cfDNA, plasma first approach, cfDNA first. *cfDNA* cell-free DNA, *IASLC* International Association for the Study of Lung Cancer, *NSCLC* non-small cell lung cancer

Liquid biopsies may also overcome tumour heterogeneity sampling bias associated with tissue biopsy and/or permit longitudinal studies of tumour evolution and response to therapy [8, 30]. A further advantage of liquid biopsy is the avoidance of invasive procedures for tissue acquisition [30]. However, there are concerns that overreliance on liquid biopsy could lead to poorer tissue pathology services in some laboratories, and the technique is not without limitations. For example, there is a lack of consensus on optimal pre-analytical procedures or consistently validated thresholds, and a scarcity of reporting guidelines. Nevertheless, new recommendations on liquid biopsy are emerging [30, 34], most notably with the updated consensus statement from the International Association for the Study of Lung Cancer (IASLC), published in 2021 (see Fig. 3) [34]. Additionally, there remains a risk of false negatives (sensitivity ~ 87%) as not all tumours shed sufficient cfDNA for detection, and cfDNA sequencing cannot distinguish morphological transition in the context of disease relapse with kinase inhibitor therapy [30]. Negative results from cfDNA analysis should therefore be confirmed by tissue testing (including a tissue re-biopsy if necessary). In the authors’ opinion, the issue of specificity will probably represent another significant limiting factor for new markers detected in cfDNA. Unlike *EGFR* mutations, which are highly specific for NSCLC, other mutations, such as *BRAF p.V600E*, are seen in different human malignancies. For these mutations, liquid biopsy can provide vital additional information to aid decision-making and may lead to the identification of a different tumour than expected, or a second tumour. Clonal haematopoiesis may also result in the expansion of mutations in peripheral blood cells, which can cause false positives if the liquid biopsy results are misinterpreted [29]. Finally, challenges limit liquid biopsy for gene fusion analysis by means of RNA-based NGS. Tumour cell-free RNA (cfRNA) can be found in the circulation but studies evaluating cfRNA as a diagnostic tool have been hampered by poor reproducibility and specificity due to issues with isolation procedures and background noise from healthy cells. Novel strategies to preserve, extract, and sequence extracellular mRNAs from plasma may help to overcome these obstacles in the future [35]. Given the limitations at present, it is recommended to pursue tissue-based testing whenever possible, and a detailed protocol for tissue utilisation and liquid biopsy should be established in each laboratory for evaluation of predictive biomarkers [36].

### Interpretation of results from cytology specimens

Cytology specimens have been demonstrated to be suitable for genomic profiling of patients with lung cancer [37, 38]. However, as visual verification of cellularity is not always possible, in the authors’ experience, potential false negative results should be considered in the absence of detected variants. Several factors may limit the accuracy of biomarker testing from cytological specimens including the potential small number of tumour cells analysed that may not recapitulate tumour heterogeneity, low DNA/RNA input, and a low ratio of neoplastic cells to non-transformed cells.

## Reporting of biomarker results

Accurate reporting of biomarker test results is paramount for timely delivery of optimal therapy, particularly given the increasing focus on minimising the time from referral for specialist care to initiation of treatment [39]. However, the complexity of reporting has increased with the growing number of clinically relevant biomarkers and there is a need for standardisation [40–42]. Although multimarker panel reports may include information on potentially beneficial classes of treatment, the use of larger panels can identify variants of unknown significance, potentially complicating interpretation [43]. ESCAT rankings can help clinicians prioritise biomarker testing and may therefore improve interpretation [10]. Overall, a number of reporting pitfalls have been identified that hinder interpretation of test results [44]. As clinicians must communicate findings to patients and are ultimately responsible for selecting appropriate targeted therapies, it is perhaps not surprising that a 2020 survey of oncologist confidence in genomic testing found that they were more confident in using single-gene tests and less confident in using multimarker panel tests to guide patient care [45].

To address the increasing complexities associated with reporting molecular pathology findings, key reporting criteria were proposed by the International Organization for Standardization (ISO). These criteria recommend that reports include an interpretation of the results, with cautionary or explanatory notes (wherever relevant) [46]. In the authors’ opinion, the inclusion of information around potential limitations may be particularly relevant to small specimen biomarker testing in NSCLC, where the quality or adequacy of the primary sample may compromise the result or interpretation. On this basis, the authors recommend including a comment on the certainty of the diagnosis (i.e. the likelihood of false positive [e.g. presence of variants of uncertain significance or of low allelic frequency] or false negative results [due to low cellularity]). Expert group recommendations on NSCLC diagnostic procedures also advocate clinical interpretation in laboratory reports, specifically through inclusion of a statement on the probability of the cancer responding to, or resisting, a specific class of drug [39]. In support of these recommendations, a recent observational study of components currently present in NSCLC molecular pathology request forms and reports found that the reporting item considered most important by pathologists and/or molecular biologists and clinicians was the clinical interpretation of the test result; the study also proposed templates to facilitate complete reporting [44]. Reporting criteria were also recently reviewed by Kerr and colleagues [8], whose recommendations are shown in Table 3.

**Table 3:** Reporting criteria for medical laboratories, adapted from ISO 15189, and additional considerations for biomarker testing [8]

Category	Minimum ISO 15189 criteria	Additional considerations for biomarker testing
General	• Results should be reported accurately, clearly, unambiguously, and in accordance with specific procedural instructions • The laboratory should define the format and medium of the report and the manner in which it is to be communicated • The laboratory should have a procedure to ensure the correctness of transcription of laboratory results • The laboratory should have a process for notifying the requester when an examination is delayed	• Molecular test data should be reported in the context of the histo/cytopathology findings so that clinical relevance is assured • Provide the report within 5–10 working days • Test results should be discussed at the MDTB/MTB
Report attributes	• Comment on sample quality that might compromise examination results • Comment on sample suitability with respect to acceptance/rejection criteria • Include critical results • Interpret comments on results	• Include a statement around the probability of the cancer responding to (or resisting) targeted therapy^a^ and/or recommendation for discussing the results at the MDTB/MTB
Report content	• Include a clear, unambiguous identification of the examination including, where appropriate, the examination procedure • Identify the laboratory that issued the report • Identify all examinations that have been performed by a referral laboratory • State the type of primary sample and date of collection • State the measurement procedure^b^ • Examination results should be reported in SI units, units traceable to SI units, or other applicable units • State biological reference intervals, clinical decision values, or include diagrams/nomograms supporting clinical decision values^b^ • Include interpretation of results, where appropriate • Identify examinations undertaken as part of a research or development programme	• Include a description of the material used for analysis including pre-analytical parameters such as fixative and fixation time, tumour cell enrichment method and final neoplastic cell content and/or amount of DNA • State the analytical technology used, details of tests used, known limitations of tests and corresponding positive/negative predictive values if published

Table adapted from Kerr et al. [8]. Copyright © 2021 The Authors. Published by Elsevier B.V. All rights reserved. Reproduced under the terms of Creative Commons Attribution 4.0 International (CC BY 4.0) license

*ISO* International Organization for Standardization, *MDTB* multidisciplinary tumour board, *MTB* molecular tumour board

^a^Where applicable; countries may vary with respect to treatment guidance

^b^Where applicable

As highlighted in the ISO requirements, complete interpretation of laboratory results may require clinical context that is not available within the laboratory [46]. In these instances, multidisciplinary teams comprising healthcare professionals from different clinical specialties are fundamental to the interpretation of complex genetic information and work collaboratively to determine the optimal clinical management for individual patients [47, 48]. Based on the large number of actionable mutations and available targeted therapies, clinical decision-making for patients with NSCLC can be particularly challenging. In many countries, multidisciplinary tumour boards (MDTBs) comprising healthcare professionals from diverse specialties are mandatory for the management of all patients with newly diagnosed NSCLC; molecular tumour boards (MTBs) may also be required to discuss complicated cases with rare mutations or complex mutational profiles [47, 49]. In addition to interpreting molecular findings in relation to the sample quality (e.g. tumour content and risk of false negatives), it is also important to review any findings in the overall context of the tissue diagnosis. Rare subtypes of adenocarcinoma and tumours with combined histology as well as other factors may impact or explain unusual molecular findings.

While access to a local MDTB/MTB is deemed essential [39], not all patients with advanced NSCLC have access to the advice gained from these discussions. Many patients will not require discussion (for example, where one clear alteration such as *EGFR* mutation or *ALK* fusion is identified); as such, some oncologists remain sceptical about the benefits of an MDTB/MTB [50]. Some MTBs favour regional collaboration between tertiary care centres and peripheral hospitals to increase patient numbers [50]. MTBs may also operate nationally or internationally; for example, an MTB portal with automated NGS data interpretation and reporting has been established across seven European cancer centres within the Cancer Core Europe network [51]. Ultimately, the goal of an MDTB/MTB is to offer the physician recommendations on optimal and available personalised therapeutic options for individual patients.

As telemedicine is expected to continue to develop following the COVID-19 pandemic, implementation of virtual MDTBs/MTBs should be considered where appropriate, as they may help to increase the efficiency of multidisciplinary care [52]. In addition, implementation of clinical pathways for patients with metastatic NSCLC may support clinical decision-making and help manage resources [53].

## External quality assessment/control

Whatever testing modality is chosen, it is imperative that laboratories perform adequate internal and external process validation and quality assessment [1]. Participation in external quality assessment (EQA) schemes is mandatory in many countries as EQA provides objective feedback to maximise accuracy and standardisation of diagnostic testing across laboratories [39].

Multiple international and European organisations currently run EQA programmes for NSCLC, and a selection of the largest programmes is summarised in Table 4. Sources of test samples employed by EQA providers vary from artificial formalin-fixed paraffin-embedded materials from engineered human cell lines with homogenous mixtures of controlled neoplastic cell content (which allow the testing of specific ratios of mutant to wild-type alleles) to real human tumour tissue. The latter most closely reflects the challenges faced by every laboratory in the real-world setting. Following sample analysis, participating laboratories produce a written report, which—at least in some EQA programmes—is sent to the EQA provider for review and assessment. Subsequently, EQA providers issue individual feedback reports to help laboratories improve their performance [54]. EQA providers may publish the laboratory protocols of the most successful participants as a recommendation of best practice and thus help to implement corrective actions in laboratories with poor results.

**Table 4:** Summary of largest EQA programmes for NSCLC in Europe [54]

EQA provider	NSCLC targets	Link
European Society of Pathology EQA (ESP-EQA)	*EGFR, KRAS, BRAF, MET, ALK, ROS1*, PD-L1	http://lung.eqascheme.org/
EMQN CIC	*KRAS, EGFR, BRAF*	https://www.emqn.org/eqa-scheme-catalogue/
Genomics Quality Assessment (GenQA)	*EGFR*, *ALK*, *ROS1*, *KRAS*, *BRAF*, *PIK3CA*, *RET*, *MET* (amplification), *MET* (exon 14 skipping), *ERBB2/HER2* (SNVs only)	https://genqa.org/eqa
Gen&Tiss (French national EQA scheme)	*KRAS, EGFR, BRAF*	http://www.genetiss.org/
Qualitätssicherungs-Initiative Pathologie (QuIP)	*KRAS, EGFR*	https://www.quip.eu/de_DE/

*ALK* anaplastic lymphoma kinase, *BRAF* B-Raf proto-oncogene, *EGFR* epidermal growth factor receptor, *EQA* external quality assessment, *ERBB2* Erb-B2 receptor tyrosine kinase 2, *HER2* human epidermal growth factor receptor 2, *KRAS* Kirsten rat sarcoma viral oncogene homolog, *MET* hepatocyte growth factor receptor, *NSCLC* non-small cell lung cancer, *PD-L1* programmed cell death ligand 1, *PIK3CA* phosphatidylinositol-4,5-bisphosphate 3-kinase catalytic subunit alpha, *RET* rearranged during transfection, *ROS1* ROS proto-oncogene 1, *SNV* single-nucleotide variant

There are several limitations associated with EQA. The increasing genomic complexity associated with precision medicine precludes EQA of every diagnostic parameter of interest; for example, sample preparation is generally excluded [44]. Therefore, EQA is not a substitute for internal quality controls and the routine utilisation of appropriate reference materials [44]. Additionally, there is a cost associated with EQA participation, along with the resource cost to the laboratory of undertaking the testing required to take part. Furthermore, the cost of testing can be more than the participation fee if several samples are tested per year. More recently, cfDNA pilot EQA schemes have been organised by several EQA providers, such as EMQN CIC, Genomic Quality Assessment (GenQA), the European Society of Pathology (ESP) Foundation, Gen&Tiss, and the Qualitätssicherungs-Initiative Pathologie (QuIP). Additionally, the International Quality Network for Pathology (IQN Path) organised a collaborative cfDNA pilot scheme (including ESP, Associazione Italiana di Oncologica Medica [AIOM], EMQN CIC, and GenQA); results were published in 2018 [55, 56]. This pilot scheme demonstrated the importance of validating methods for cfDNA detection, and resulted in recommendations for laboratories to review their EQA results to limit the amount of analytical detection errors [55, 56] and improve the reporting of laboratory results to the referring clinician.

**Fig. 4:**
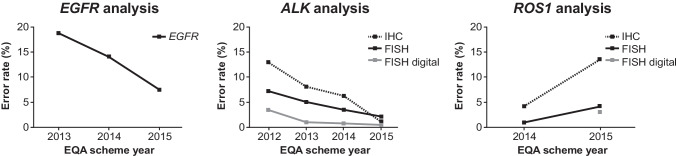
Error rates in lung cancer biomarker analysis for *EGFR*, *ALK*, and *ROS1* across EQA schemes run by the European Society of Pathology, 2012–2015 [58]. Figure adapted from Keppens et al. [58]. Copyright © 2021 The Authors. Published by Impact Journals. All rights reserved. Reproduced under the terms of Creative Commons Attribution 3.0 International (CC BY 3.0) license. *ALK* anaplastic lymphoma kinase, *EGFR* epidermal growth factor receptor, *EQA* external quality assessment, *FISH* fluorescent in situ hybridisation, *IHC* immunohistochemistry, *ROS1* ROS proto-oncogene 1

EQA schemes are particularly important when it comes to the adoption and harmonisation of novel biomarkers. There is robust evidence to demonstrate that EQA programmes play an essential role in improving biomarker detection accuracy for patients (Fig. 4).

The importance of EQA is also reflected by ISO 15189:2012 accreditation of diagnostic laboratories, which requires participation in EQA schemes [44]. In some but not all countries, EQA providers can directly pass on information on poor laboratory performance to the appropriate authorities (e.g. the National Quality Assessment Advisory Panel of the Royal College of Pathologists in the UK and Sciensano in Belgium).

## Conclusions

Given the growing number of biomarkers in lung cancer and associated tissue constraints, the testing of predictive biomarkers must follow an optimal approach to maximise diagnostic yield of limited tissue samples. This results in transition from a single-gene approach with testing of one or more individual markers to a multigene approach represented by NGS, which is more cost-effective (see Table 5 for a summary of key opinions and recommendations around key aspects of analysis, reporting, and quality assessment). NGS testing alone may be appropriate where limited tissue is available for diagnosis/molecular testing (except for PD-L1, and where IHC is the method of choice). Similarly, plasma-based NGS (notwithstanding sensitivity/specificity issues) and tissue-based methods are complementary approaches as knowledge of tissue testing outcomes aids in the interpretation of circulating cfDNA analysis. Whatever testing modality is used, adequate internal validation and quality control schemes are crucial. Participation in EQA schemes can help to ensure high levels of accuracy and standardisation of testing across laboratories. Finally, accurate, detailed reporting with interpretation of test results, as facilitated by MDTBs/MTBs, can help ensure the timely delivery of optimal treatment selection for patients with advanced NSCLC.

**Table 5:** A summary of recommendations around key aspects of analysis, reporting, and quality assessment

Key opinions and recommendations^a^
***Biomarker testing methodologies*** *Multiplex and single-gene testing* • NGS is more cost-effective than single-gene testing when multiple targets need to be tested [16–18] • Combined DNA/RNA NGS is a reliable and efficient approach for comprehensive detection of all approved and emerging biomarkers in advanced NSCLC (excluding PD-L1 detection by IHC) • RNA-based NGS in parallel with DNA-based NGS offers improved sensitivity for the detection of gene fusions • RNA-based NGS allows identification of gene transcripts, permitting conclusions regarding in-frame gene fusions and identification of gene fusion partners • One-step co-extraction of RNA and DNA and simultaneous NGS of both DNA and RNA can help reduce tissue consumption [23, 24] • Hybrid capture assay and anchored multiplex technology allow broader fusion analysis but require a larger amount of material than amplicon-based methods [22] *IHC testing for gene fusions* • IHC may be complementary to, and/or an alternative to, sequencing or FISH testing • Detection of gene-product overexpression by IHC is a useful screening tool for assessing *ALK*, *ROS1*, and *NTRK* fusions in NSCLC • For ROS1 and NTRK IHC + cases, confirmation by another molecular method (e.g. FISH, qPCR, NGS) is mandatory according to ESMO guidelines [1] • For RET fusions, IHC is not recommended as a screening tool, as false positive and negative cases have been reported [26] *Liquid and tissue biopsy* • Sequencing of plasma-circulating cfDNA via liquid biopsy is a complementary approach to tissue-based biomarker testing [29] • cfDNA sequencing analysis can be conducted using as little as 6 mL of peripheral whole blood stored at room temperature in EDTA tubes • Blood collected in EDTA tubes should be centrifuged within 3 h to reduce degradation of cfDNA and the risk of a false negative result • Analytical techniques must be highly sensitive to detect tumour-specific cfDNA, which represents only a small fraction of total circulating cfDNA • Limited data exist on the use of alternative biological fluids for liquid biopsy in the genomic characterisation of NSCLC for guiding therapy • Given the current limitations of liquid biopsies (e.g. false negatives), tissue-based testing should be pursued whenever possible, and a detailed protocol for tissue utilisation and liquid biopsy should be established in each laboratory for evaluation of predictive biomarkers [36] • Negative results from cfDNA analysis should be confirmed by tissue testing (including a tissue re-biopsy if necessary) due to variability in tumour DNA shedding and the high risk of false negatives • Positive results from cfDNA analysis should be considered with caution due to the potential for false positives attributable to clonal haematopoeisis and other factors *Cytological specimens* • Cytology specimens can be suitable for genomic profiling of patients with lung cancer [37, 38]. However, as visual verification of cellularity is not always possible, potential false negative results should be considered in the absence of detected variants
***Reporting of biomarker results*** • Accurate reporting of biomarker test results is paramount for timely delivery of optimal therapy • ESCAT rankings can help prioritise biomarker testing and may therefore improve interpretation [10] • Key criteria proposed by the International Organization for Standardization (ISO) should be reported and include an interpretation of the results, with cautionary or explanatory notes (wherever relevant) [46] • A comment on the certainty of the diagnosis (i.e. the likelihood of false positive [e.g. presence of variants of uncertain significance or of low allelic frequency] or false negative results [due to low cellularity]) is recommended • A statement on the probability of the cancer responding to, or resisting, a specific class of drug is recommended by the European Expert Group on diagnostic procedures for NSCLC [39] • Multidisciplinary teams comprising healthcare professionals from different clinical specialties are fundamental to the interpretation of complex genetic information [47, 48]
***External quality assessment/control*** • It is imperative that laboratories perform adequate internal and external process validation and quality assessment [1] • Participation in EQA schemes is mandatory in many countries as EQA provides objective feedback to maximise accuracy and standardisation of diagnostic testing across laboratories [39]

^a^Where no guidelines or literature explicitly describe best practice, recommendations for best practice are reported according to the experience of the author group

*ALK* anaplastic lymphoma kinase, *cfDNA* cell-free DNA, *DNA* deoxyribonucleic acid, *EQA* external quality assessment, *ESCAT* ESMO Scale for Clinical Actionability of molecular Targets, *ESMO* European Society for Medical Oncology, *EDTA* ethylenediaminetetraacetic acid, *IHC* immunohistochemistry, *NGS* next-generation sequencing, *NSCLC* non-small cell lung cancer, *NTRK* neurotrophic tyrosine receptor kinase, *PD-L1* programmed death ligand 1, *RET* rearranged during transfection, *RNA* ribonucleic acid, *ROS1* ROS proto-oncogene 1

## Data Availability

Data sharing not applicable. No new data were created or analysed in this article.
